# The complete chloroplast DNA sequences of the charophycean green algae *Staurastrum* and *Zygnema* reveal that the chloroplast genome underwent extensive changes during the evolution of the Zygnematales

**DOI:** 10.1186/1741-7007-3-22

**Published:** 2005-10-20

**Authors:** Monique Turmel, Christian Otis, Claude Lemieux

**Affiliations:** 1 Département de Biochimie et de Microbiologie, Université Laval, Québec, Québec, G1K 7P4, Canada

## Abstract

**Background:**

The Streptophyta comprise all land plants and six monophyletic groups of charophycean green algae. Phylogenetic analyses of four genes from three cellular compartments support the following branching order for these algal lineages: Mesostigmatales, Chlorokybales, Klebsormidiales, Zygnematales, Coleochaetales and Charales, with the last lineage being sister to land plants. Comparative analyses of the *Mesostigma viride *(Mesostigmatales) and land plant chloroplast genome sequences revealed that this genome experienced many gene losses, intron insertions and gene rearrangements during the evolution of charophyceans. On the other hand, the chloroplast genome of *Chaetosphaeridium globosum *(Coleochaetales) is highly similar to its land plant counterparts in terms of gene content, intron composition and gene order, indicating that most of the features characteristic of land plant chloroplast DNA (cpDNA) were acquired from charophycean green algae. To gain further insight into when the highly conservative pattern displayed by land plant cpDNAs originated in the Streptophyta, we have determined the cpDNA sequences of the distantly related zygnematalean algae *Staurastrum punctulatum *and *Zygnema circumcarinatum*.

**Results:**

The 157,089 bp *Staurastrum *and 165,372 bp *Zygnema *cpDNAs encode 121 and 125 genes, respectively. Although both cpDNAs lack an rRNA-encoding inverted repeat (IR), they are substantially larger than *Chaetosphaeridium *and land plant cpDNAs. This increased size is explained by the expansion of intergenic spacers and introns. The *Staurastrum *and *Zygnema *genomes differ extensively from one another and from their streptophyte counterparts at the level of gene order, with the *Staurastrum *genome more closely resembling its land plant counterparts than does *Zygnema *cpDNA. Many intergenic regions in *Zygnema *cpDNA harbor tandem repeats. The introns in both *Staurastrum *(8 introns) and *Zygnema *(13 introns) cpDNAs represent subsets of those found in land plant cpDNAs. They represent 16 distinct insertion sites, only five of which are shared by the two zygnematalean genomes. Three of these insertions sites have not been identified in *Chaetosphaeridium *cpDNA.

**Conclusion:**

The chloroplast genome experienced substantial changes in overall structure, gene order, and intron content during the evolution of the Zygnematales. Most of the features considered earlier as typical of land plant cpDNAs probably originated before the emergence of the Zygnematales and Coleochaetales.

## Background

About 450 million years ago, green algae belonging to the class Charophyceae emerged from their aquatic habitat to colonize the land [1-3]. This important event in the history of life gave rise to all the land plant species that make up the flora of our planet. The few thousand species of charophycean green algae that are alive today exhibit great variability in cellular organization and reproduction [4]. With the land plants, they form the green plant lineage Streptophyta [5], whereas all other green algae (more than 10,000 species), with perhaps the exception of *Mesostigma viride*, belong to the sister lineage Chlorophyta [4]. Five monophyletic groups of charophycean green algae have been recognized: the Chlorokybales, Klebsormidiales, Zygnematales, Coleochaetales and Charales [6], given here in order of increasing cellular complexity. *Mesostigma *may represent an additional lineage of the Charophyceae, the Mesostigmatales, as indicated by phylogenetic studies that placed this unicellular green alga at the base of the Streptophyta [7-10]. This lineage, however, remains controversial, considering that separate analyses based on a large number of chloroplast- or mitochondrial-encoded proteins [11-13] and on the chloroplast small and large subunit rRNA genes [14] identified *Mesostigma *before the divergence of the Chlorophyta and Streptophyta.

On the basis of morphological characters alone, the two charophycean groups that exhibit the greatest cellular complexity, *i.e*. the Charales and Coleochaetales, have been proposed to be the closest relatives of land plants [15,16]. Recent analyses of the combined sequences of four genes from the nucleus (small subunit rRNA gene), chloroplast (*atpB *and *rbcL*) and mitochondria (*nad5*) of 25 charophycean green algae and eight green plants revealed that the Charales and land plants form a highly supported clade; however, moderate bootstrap support was observed for the positions of the other charophycean groups [8]. The best trees inferred by Bayesian and maximum likelihood methods in this four-gene analysis support an evolutionary trend toward increasing cellular complexity [17]. In contrast, all phylogenies of charophycean green algae previously inferred from a smaller number of genes failed to provide any conclusive results concerning the branching order of the charophycean green algae and their relationships with land plants [15,16].

We have recently undertaken the sequencing of complete chloroplast genomes from representatives of the various charophycean lineages in order to elucidate the branching order of these lineages and also to understand the evolution of chloroplast DNA (cpDNA) within the Streptophyta. We have reported thus far the cpDNA sequences of *Mesostigma *(Mesostigmatales) [11] and *Chaetosphaeridium globosum *(Coleochaetales) [18]. Comparative analyses of the *Mesostigma *cpDNA sequence (136 genes, no introns) with its land plant counterparts (110–120 genes, about 20 introns) revealed that the chloroplast genome underwent substantial changes in its architecture during the evolution of streptophytes (namely gene losses, intron insertions and scrambling of gene order). At the levels of gene content (125 genes), intron composition (18 introns) and gene order, *Chaetosphaeridium *cpDNA is remarkably similar to land plant cpDNAs, implying that most of the features characteristic of land plant lineages were acquired from charophycean green algae. Like the cpDNAs of many chlorophytes, those of *Mesostigma*, *Chaetosphaeridium *and most land plant species exhibit a quadripartite structure that is characterized by the presence of two copies of a rDNA-containing inverted repeat (IR) separated by large and small single-copy regions. All the genes they have in common, with a few exceptions, reside in corresponding genomic regions.

In this study, we report the complete cpDNA sequences of two members of the Zygnematales that belong to distinct lineages, *Staurastrum punctulatum *and *Zygnema circumcarinatum*. Although the chloroplast genomes of these charophycean green algae closely resemble their *Chaetosphaeridium *and bryophyte counterparts at the primary sequence and gene content levels, they feature substantial differences at the levels of structure, gene order and intron content. Like the cpDNA of the zygnematalean alga *Spirogyra maxima *[19], both *Staurastrum *and *Zygnema *cpDNAs lack a large IR. Clearly, loss of the IR appears to be a major event that shaped the architecture of the chloroplast genome in the Zygnematales, an event that apparently occurred early during the evolution of this group of charophycean green algae.

## Results

### Selection of taxa

The Zygnematales as circumscribed by Bold and Wynne [20] comprise the green algae whose mode of sexual reproduction is conjugation. This is the most important charophycean lineage in terms of diversity and number of species (~50 genera and ~6,000 species) [16]. Classification schemes based on cell wall organization have recognized two groups of conjugating green algae: first, the unicellular or multicellular green algae with an ornamented and segmented cell wall, also called placoderm desmids and often treated as members of the order Desmidiales, and second, the green algae that bear a smooth cell wall, which are often classified separately in the Zygnematales [21]. Among the latter group are found filamentous forms and the saccoderm desmids that consist either of unicells or loosely joined cells. Phylogenies inferred using *rbcL *[21] or the combined *rbcL *and nuclear small subunit rRNA genes [22] support the monophyly of placoderm desmids and place the filamentous and saccoderm desmids together in a distinct monophyletic group. For our study, we have selected a representative of each of these two monophyletic groups: *Staurastrum *is a unicellular, placoderm desmid, whereas *Zygnema *is a filamentous green alga with a non-ornamented cell wall.

### General features

**Figure 1:**
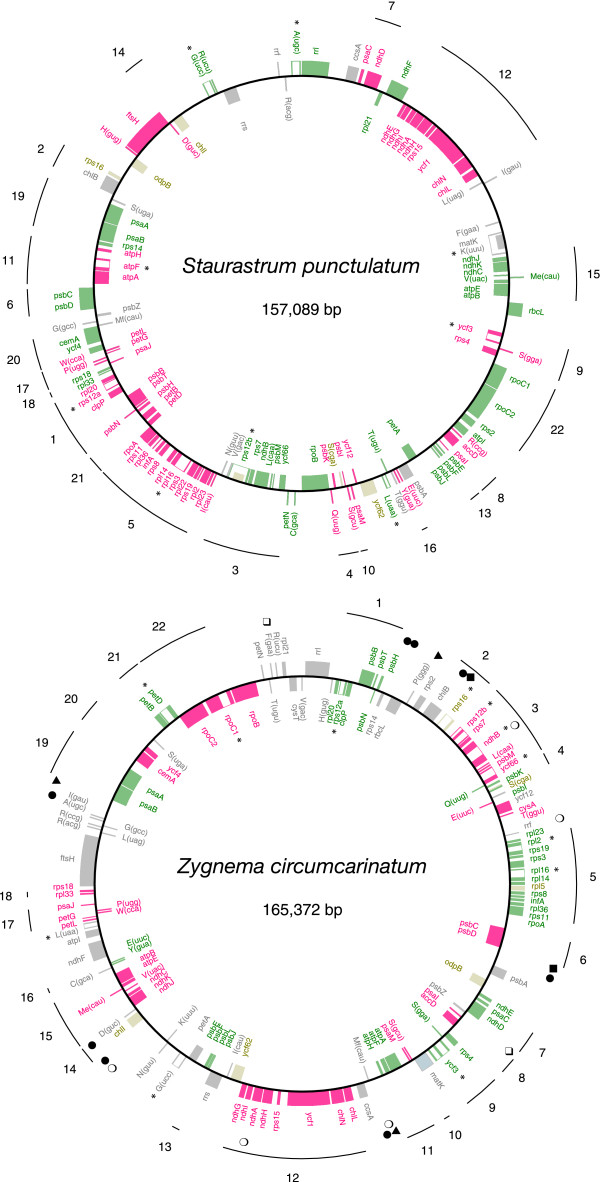
**Gene maps of *Staurastrum *and *Zygnema *cpDNAs**. Genes (filled boxes) shown on the outside of each map are transcribed in a clockwise direction, whereas those on the inside of each map are transcribed counterclockwise. Genes absent from *Marchantia *cpDNA are represented in beige. Gene clusters shared with *Marchantia *cpDNA [GenBank:NC_001319] are shown as alternating series of green and red boxes. Genes present in *Marchantia *cpDNA but located outside conserved clusters are shown in grey. Gene clusters shared by the two zygnematalean cpDNAs are represented by labelled bars outside each map. Genes containing introns (open boxes) are denoted by asterisks. Dispersed repeat regions in *Zygnema *cpDNA that contain short tandem repeats are denoted by symbols. The repeat units in these regions are as follows: filled squares, TAGAA; open squares, TTCTA; filled circles, GTAT; open circles, ATAC; filled triangles, CTTA. Note that filled and open symbols with the same geometric shape represent the repeat regions of which the sequences are in inverted orientation relative to one another. The intron sequences bordering the *rps12 *exons (*rps12a *and *rps12b*) are spliced in *trans *at the RNA level. tRNA genes are indicated by the one-letter amino acid code (Me, elongator methionine; Mf, initiator methionine) followed by the anticodon in parentheses. The ORFs unique to *Staurastrum *or *Zygnema *cpDNA are not indicated (see [GenBank:AY958085] and [GenBank:AY958086] for more details).

The 157,089-bp *Staurastrum *[GenBank:AY958085] and 165,372-bp *Zygnema *[GenBank:AY958086] cpDNAs map as circular molecules containing 121 and 125 genes, respectively (Fig. 1). Both genomes lack a rDNA-containing IR and no remnant of such a sequence could be detected during our analysis of repeated elements. All genes are present in single copy, with the exception of the duplicated *Zygnema trnE(uuc) *gene, the sequences of which differ at two positions. Note that the *matK *gene was not included in the total number of genes calculated for *Zygnema *cpDNA, because this gene occurs as an intron ORF in all other streptophytes where it has been identified. Aside from the absence of the IR, the most prominent differences displayed by the two zygnematalean cpDNAs relative to their counterparts in *Chaetosphaeridium *[18] and land plants (here represented by the bryophyte *Marchantia polymorpha *[23]) are their larger size (taking into consideration the absence of the IR from these genomes) and their smaller number of *cis*-spliced group II introns (Table 1). The larger size of zygnematalean cpDNAs is mainly explained by the expansion of intergenic spacers (Table 2). The latter sequences represent 42% of the genome in both *Staurastrum *and *Zygnema *cpDNAs compared to about 20% in *Chaetosphaeridium *and land plant cpDNAs. Introns have also expanded in size in both zygnematalean cpDNAs compared to their *Chaetosphaeridium *and land plant homologues (Table 2).

### Gene content

Table 3 compares the gene contents of *Staurastrum, Zygnema, Chaetosphaeridium *and *Marchantia *cpDNAs. The two zygnematalean cpDNAs share 120 genes, 116 of which are present in both *Chaetosphaeridium *and *Marchantia *cpDNAs. Five genes in *Zygnema *cpDNA are missing from *Staurastrum *cpDNA; they encode the tRNA^Pro^(GGG), tRNA^Ser^(CGA), ribosomal protein L5, and the proteins CysA and CysT that are involved in sulfate transport. Although there is no functional *trnS(cga) *in *Staurastrum *cpDNA, a *trnS(cga) *pseudogene was identified in this genome. A standard acceptor stem could not be modelled from the RNA sequence derived from this pseudogene; the 5' region of this sequence diverges considerably from homologous tRNA sequences in other streptophytes and cannot base pair with the 3' region. *Staurastrum *exhibits only one chloroplast gene (*rpl22*) that is missing from *Zygnema*. To our knowledge, this is the first time that the loss of *rpl22 *together with that of *rpl32 *(a gene absent from both zygnematalean cpDNAs) has been reported in the Streptophyta. As in land plant cpDNAs, but in contrast to *Chaetosphaeridium *cpDNA, no *tufA*-like sequence was detected in the two zygnematalean cpDNAs. It appears that only the *chlI*, *odpB *and *ycf62 *genes were specifically lost just before or concurrently with the emergence of land plants (Table 3). Note that the *rps16 *gene cannot be included in this category, as it is present in the majority of land plant cpDNAs sequenced to date.

### Gene order

*Staurastrum *and *Zygnema *cpDNAs differ substantially from one another and from their *Chaetosphaeridium *and land plant counterparts at the level of gene organization (Table 4). Eighty-two genes in the two zygnematalean cpDNAs form 22 blocks of colinear sequences, which are highly scrambled in order (Fig. 1). A minimum of 59 inversions would be required to convert the gene order of *Staurastrum *cpDNA into that of *Zygnema *cpDNA (Table 4).

**Figure 2:**
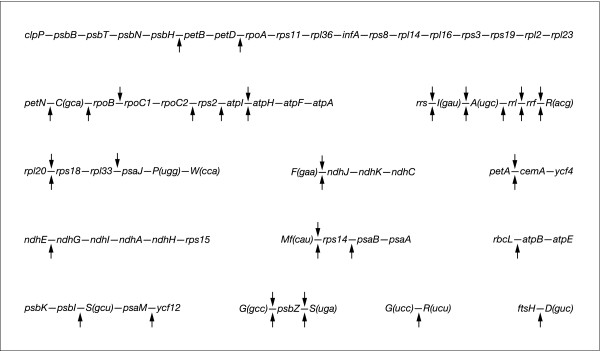
**Fragmentation of ancestral chloroplast gene clusters during the evolution of the Zygnematales**. The ancestral clusters shown are found in both *Mesostigma *[GenBank:NC_002186] and *Marchantia *[GenBank:NC_001319] cpDNAs. The top and bottom arrows denote the sites where they are broken in *Staurastrum *and *Zygnema *cpDNAs, respectively. For the polarities of the genes relative to one another, the reader should consult the gene map of *Mesostigma *cpDNA [11].

Of the two zygnematalean cpDNAs, that showing the most similar gene arrangement with its *Chaetosphaeridium *and land plant counterparts is *Staurastrum *cpDNA (Table 4). In both *Staurastrum *and *Zygnema *cpDNAs, the gene organization more closely resembles that of *Marchantia *than that of *Chaetosphaeridium *(Table 4). *Staurastrum *cpDNA shares with its *Marchantia *counterpart 22 blocks of colinear sequences that contain a total of 101 genes, whereas *Zygnema *cpDNA shares 20 blocks featuring 81 genes (Fig. 1). Close inspection of these blocks relative to those conserved between *Mesostigma *and *Marchantia *cpDNAs [11] reveals that 13 ancestral gene clusters, including those containing the rDNA, *atpA*, *psbB *and *rpoB *operons, were fragmented at 27 sites during the evolution of the Zygnematales (Fig. 2). Eleven of these rearrangement breakpoints are common to the two green algal cpDNAs, whereas 2 and 14 breakpoints are unique to *Staurastrum *and *Zygnema *cpDNAs, respectively. Assuming that these unique rearrangement breakpoints appeared after the divergence of the two zygnematalean species, we infer that the chloroplast genome of the common ancestor of *Staurastrum *and *Zygnema *shared a number of derived gene clusters with *Chaetosphaeridium *and land plants. For example, the cluster of 29 genes extending from *petL *to *trnI(cau) *in *Marchantia *cpDNA and that of 13 genes delimited by *rps12b *and *atpI *were likely present in the common ancestor of *Staurastrum *and *Zygnema*. Only four gene clusters are shared specifically between zygnematalean and *Marchantia *cpDNAs: *rps4-trnS(gga)-ycf3 *(cluster 9 in Fig. 1), *atpB-atpE-trnV(uac)-trnMe(cau)-ndhC-ndhK-ndhJ *(cluster 15), *trnH(gug)-ftsH*-*trnD(guc) *(in *Staurastrum *only), and *trnE(uuc)-cysA*-*trnT(ggu) *(in *Zygnema *only).

**Figure 3:**
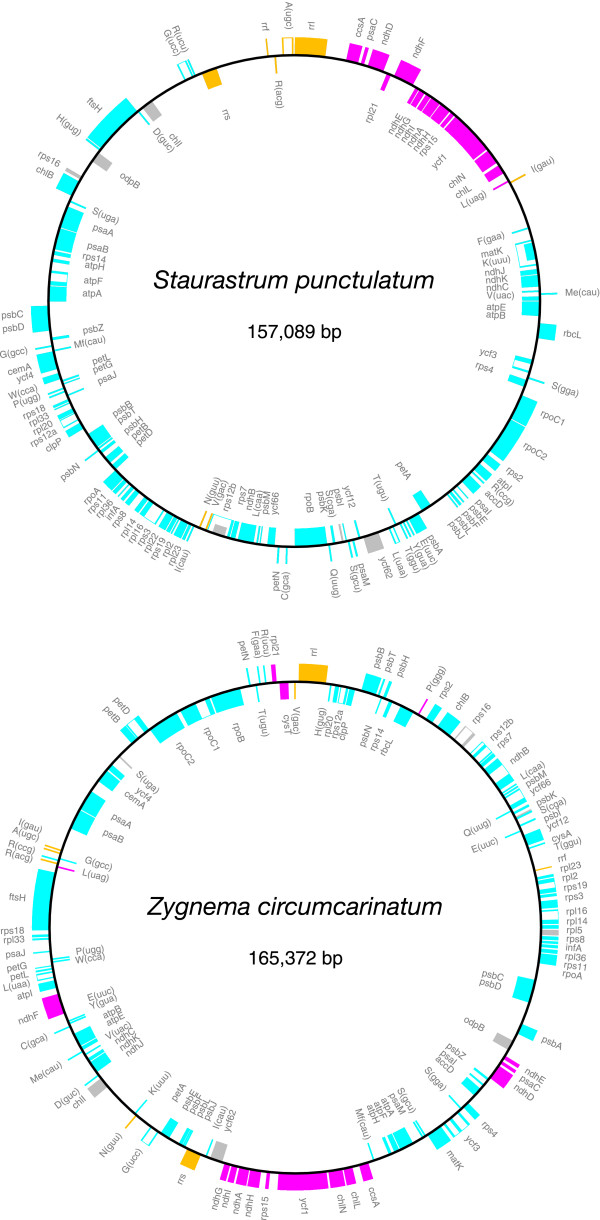
**Compared patterns of gene partitioning in zygnematalean and *Marchantia *cpDNAs**. Each gene in *Staurastrum *and *Zygnema *cpDNAs is colour-coded according to the region of *Marchantia *cpDNA [GenBank:NC_001319] carrying its homologue; cyan, large single-copy region; magenta, small single-copy region; and yellow, IR. Genes shown in grey are absent from *Marchantia *cpDNA.

The higher degree of ancestral characters displayed by *Staurastrum *cpDNA compared to its *Zygnema *homologue at the gene organizational level is also evident when one examines the genomic region in which each gene locus would be expected to map if the IR had been retained (Fig. 3). In *Staurastrum *cpDNA, the 15 genes predicted to have been present in the small single-copy region occupy a discrete region just beside five of the eight genes that usually make up the IR; in *Zygnema *cpDNA, however, the genes usually located in the small single-copy region and the IR are more widely dispersed in the genome.

### Intron composition

**Figure 4:**
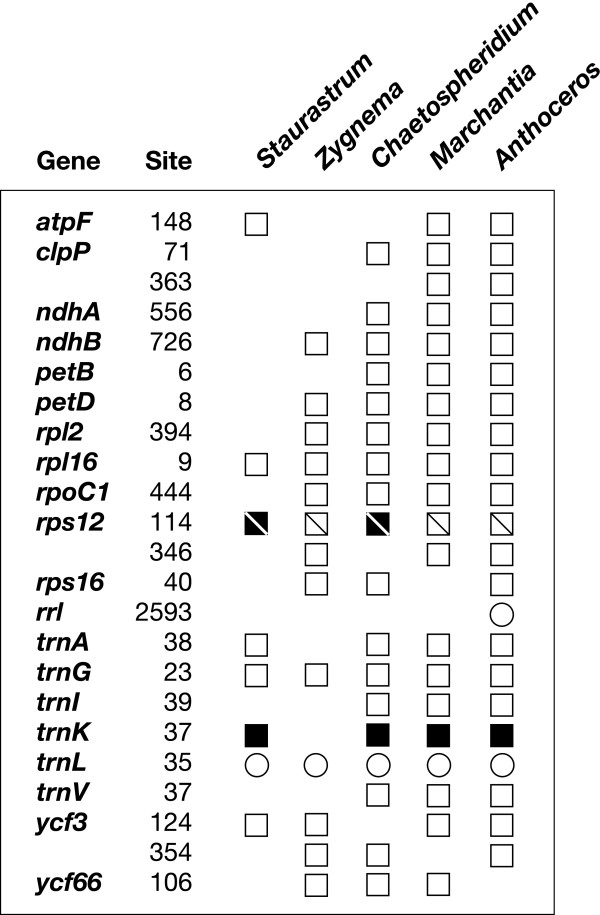
**Distributions of introns in streptophyte cpDNAs**. Circles denote the presence of group I introns, and squares denote the presence of group II introns. Divided squares represent *trans*-spliced group II introns. Open symbols denote the absence of intron ORFs, whereas filled symbols denote their presence. Intron insertion sites in protein-coding and tRNA genes are given relative to the corresponding genes in *Mesostigma *cpDNA; the insertion site in *rrl *is given relative to the *Escherichia coli *23S rRNA. For each insertion site, the position corresponding to the nucleotide immediately preceding the intron is reported. Note that *rps16 *is lacking in *Marchantia *cpDNA and that the *rrl *intron at position 2593 is absent from all completely sequenced land plant cpDNAs, with the exception of *Anthoceros *cpDNA. The intron data were taken from the following accession numbers: *Staurastrum*, [GenBank:AY958085]; *Zygnema*, [GenBank:AY958086]; *Chaetosphaeridium*, [GenBank:NC_004115]; *Marchantia*, [GenBank:NC_001319]; and *Anthoceros formosae *[GenBank:NC_004543].

As in *Chaetosphaeridium *cpDNA, the introns in *Staurastrum *and *Zygnema *cpDNAs represent subsets of those found in land plant cpDNAs (Fig. 4). Both zygnematalean cpDNAs share with their *Chaetosphaeridium *and land plant counterparts one group I intron in *trnL(uaa)*, two *cis*-spliced group II introns in *rpl16 *and *trnG(ucc)*, and one *trans*-spliced group II intron in *rps12*. Only three group II introns in *Staurastrum *and/or *Zygnema *cpDNAs (in *atpF, rps12 *at site 346 and *ycf3*) have no homologues in *Chaetosphaeridium *cpDNA. Evidence for a charophycean green algal origin of land plant group II introns is lacking for only the *clpP *intron at site 363. The *Staurastrum trans*-spliced *rps12 *intron resembles its *Chaetosphaeridium *homologue in exhibiting a large ORF in domain IV. The putative protein of 404 amino acids encoded by the *Staurastrum *ORF is related to reverse transcriptases, whereas the smaller protein (247 amino acids) specified by the *Chaetosphaeridium *ORF lacks similarity with such proteins.

**Figure 5:**
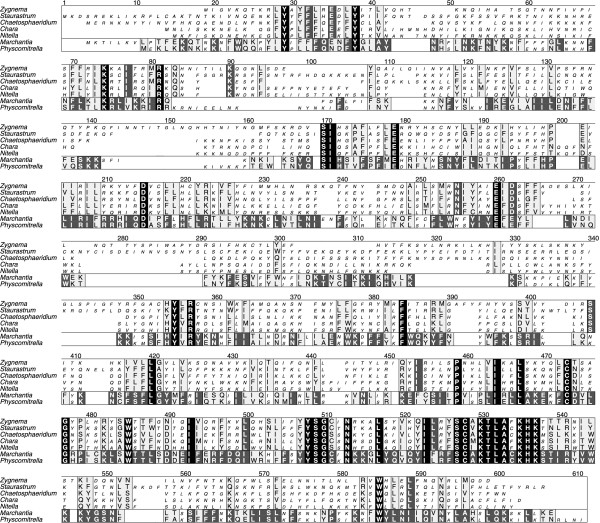
**Sequence conservation among streptophyte MatK proteins**. The MatK sequences of selected green algae and land plants were aligned with T-COFFEE [40] and arranged into two separate groups. Identical amino acids in all the sequences examined are displayed on a black background, whereas identical amino acids in all the green algal or land plant sequences are shown on a dark grey background. In each group, sets of residues sharing eight of the 10 features in the property matrix of AMAS [41] are shown on a light grey background. The accession numbers for the MatK sequences analyzed are as follows: *Zygnema*, [GenBank:AY958086]; *Staurastrum*, [GenBank:AY958085]; *Chaetosphaeridium*, [GenBank:NC_004115]; *Chara connivens*, [GenBank:AY170442]; *Nitella opaca*, [GenBank:AY170449]; *Marchantia*, [GenBank:NC_001319]; and *Physcomitrella patens *[GenBank:NC_005087].

Like its *Chaetosphaeridium *and land plant counterparts, the *cis*-spliced group II intron in *Staurastrum trnK(uuu) *encodes the maturase MatK. As mentioned earlier, a freestanding *matK *gene was identified in *Zygnema *cpDNA even though an intron is absent from *trnK(uuu) *in this charophycean green alga. Close inspection of the regions immediately flanking the *Zygnema matK *gene for the presence of sequences conserved in domains V and VI of group II introns failed to reveal any evidence that this gene had once been an integral part of a group II intron. The *Zygnema matK *is most probably a functional gene because its predicted protein features the vast majority of the conserved amino acids that the *trnK *intron-encoded MatK of *Staurastrum *shares with its *Chaetosphaeridium*, *Chara, Nitella *and land plant homologues (Fig. 5).

### Repeated sequences

Comparison of each zygnematalean cpDNA sequence against itself using PipMaker [24] indicated the presence of repeats in many intergenic regions of *Zygnema *cpDNA and the virtual absence of such sequences from *Staurastrum *cpDNA. Analysis of the *Zygnema *genome sequence with REPuter [25] revealed that the great majority of the repeat regions larger than 30 bp are composed of short tandem repeats. Each of the 35 repeat regions identified consists of 4 to 16 bp units that are repeated in tandem 4 to 50 times (Table 5). Most regions (29/35) feature repeat units of 4 or 5 bp, and the regions with GTAT, ATAC, TAGAA, TTCTA and CTTA units occur at more than one location on the chloroplast genome (Fig. 1). All three regions carrying the CTTA units feature sequences that are in direct orientation relative to one another; however, the 13 regions with the GTAT and complementary ATAC units and the four regions with the TAGAA and complementary TTCTA units form a population of dispersed repeats that are in direct or inverted orientation relative to one another. Eighty percent of the repeat regions (28/35) reside outside the blocks of sequences that are colinear with *Staurastrum *cpDNA. We estimate that at least 2,245 bp of *Zygnema *cpDNA, *i.e*. about 60% of the increased size of the *Zygnema *intergenic regions compared to their *Staurastrum *homologues, are accounted for by short tandem repeats.

Only two loci of the *Staurastrum *chloroplast genome contain short tandem repeats: a region composed of four units of the GAATAAATA sequence in the *infA*-*rpl36 *spacer and a region containing nine units of the GTATTT sequence in the *rps16-odpB *spacer. Aside from two copies of 45-bp sequence (in the *atpF-atpH *and *atpH-rps14 *spacers) that are in direct orientation, no dispersed repeats larger than 30 bp were detected in *Staurastrum *cpDNA.

## Discussion

Although *Staurastrum *and *Zygnema *cpDNAs bear high similarity in primary sequence and gene content to their *Chaetosphaeridium *and land plant counterparts, they differ substantially from one another and from the latter genomes in overall structure, gene order and intron content. From our comparative analysis of streptophyte cpDNAs, we infer that the chloroplast genome of the last common ancestor of *Staurastrum *and *Zygnema *probably lacked a large IR encoding the rRNA genes, had a low gene density, and more closely resembled *Chaetosphaeridium *and land plant cpDNAs at the gene organizational and intron levels than do *Zygnema *and *Staurastrum *cpDNAs. At least 16 of the 22 intron positions commonly found in land plant cpDNAs, including three sites that have not been identified in *Chaetosphaeridium*, were probably present in the common ancestor of *Staurastrum *and *Zygnema*.

Considering the absence of an rDNA-encoding IR region in both *Staurastrum *and *Zygnema *cpDNAs, it is not surprising that these genomes are considerably rearranged relative to their coleochaetalean and land plants counterparts that have retained the quadripartite structure. All green plant cpDNAs that have lost the IR tend to be highly scrambled in gene order [26,27]. It has been hypothesized that the loss of the IR enhances opportunities for intramolecular recombination between small dispersed repeats [28]. In agreement with the idea that there is a direct link between the frequency of intramolecular recombination events and the abundance of small dispersed repeats [28], we identified more rearrangements in the repeat-rich genome of *Zygnema *than in the repeat-poor genome of *Staurastrum*. As in the cpDNAs of the nonphotosynthetic, parasitic flowering plant *Epifagus virginiana *[29] and the evening primrose *Oenothera *[30], the repeated sequences in *Zygnema *cpDNA consist essentially of tandem repeats that probably arose by replication slippage.

A single event of IR loss likely accounts for the absence of a quadripartite structure from both *Staurastrum *and *Zygnema *cpDNAs. This hypothesis is more parsimonious than the alternative scenario involving two independent losses, and is consistent with previous evidence that the cpDNA of *Spirogyra *(a distant relative of *Zygnema*) has no IR [19]. It is also supported by our finding that *Staurastrum *and *Zygnema *cpDNAs share 11 rearrangement breakpoints within ancestral gene clusters. Given the close connection between IR loss and gene rearrangements, several of these shared breakpoints might have appeared following the loss of the IR in the lineage leading to the last common ancestor of *Staurastrum *and *Zygnema*. Considering that this ancestor occupies a basal position in the tree describing the relationships among zygnematalean green algae [21,22], then most, if not all, of the algae belonging to the Zygnematales are expected to lack an IR in their chloroplast genome.

As introns appear to be generally stable in land plant cpDNAs [28], the important difference in intron content displayed by *Staurastrum *and *Zygnema *cpDNAs is unexpected. The two zygnematalean cpDNAs share only five of the 16 intron insertion sites they exhibit in total.*Staurastrum *cpDNA lacks seven of the 13 introns that are present in *Zygnema *cpDNA, whereas the latter cpDNA lacks five of the eight introns found in the former genome. The intron distributions in these cpDNAs are best explained by assuming that all 16 insertion sites were populated with introns in the common ancestor of *Staurastrum *and *Zygnema *and that subsequently, several introns were specifically lost in each of the lineages leading to these green algae. Obviously, we cannot exclude the possibility that chloroplast introns occupying common insertion sites were lost independently in the *Staurastrum *and *Zygnema *lineages; thus, the predicted number of introns in the common ancestor of these algae may represent a minimal estimate. Given that intron losses are thought to result from insertions, through homologous recombination, of intron-less cDNA copies generated by reverse transcription [31], the frequency of homologous recombination events or the level of reverse transcriptase activity might be higher in the chloroplasts of conjugating green algae than in land plant chloroplasts. In this respect, it is interesting to note that the *Staurastrum trans*-spliced *rps12 *intron specifies a reverse transcriptase and is the only known streptophyte chloroplast intron encoding such an activity.

Our finding that *matK *is free-standing in *Zygnema *cpDNA together with the absence of the *trnK(uuu) *intron in which it usually resides strongly suggests that its putative maturase product is essential for the splicing of group II introns other than the *trnK(uuu) *intron. Circumstantial evidence that MatK functions in splicing of multiple introns has previously been reported for land plant chloroplasts. The *matK *gene is located within the group II intron of *trnK(uuu) *in all photosynthetic land plants, but occurs as a free-standing gene in *Epifagus *cpDNA [29]. *In vivo *splicing analyses of the complete set of chloroplast group II introns in land plant mutants lacking chloroplast ribosomes disclosed specific splicing defects involving mainly group IIA introns (in *atpF*, *rpl2*, *rps12*, *trnA*, *trnI*, *trnK*), thus implying that cpDNA-encoded protein(s) act as splicing factors [32-35]. It has been proposed that MatK evolved from a *trnK(uuu) *intron-specific maturase to a more versatile maturase that assists the splicing of most or all group IIA introns of land plants [32-35].

## Conclusion

Our structural analyses of the *Staurastrum *and *Zygnema *chloroplast genomes have revealed that many of the features considered earlier as typical of land plant cpDNAs originated before the emergence of the Coleochaetales and Zygnematales. While the chloroplast genome appears to have remained relatively stable in the coleochaetalean lineage, it has lost the IR and has undergone many changes in gene order and intron content during the evolution of the Zygnematales.

## Methods

### DNA isolation and cloning

Chloroplast DNA fractions from *Staurastrum punctulatum *de Brébisson (SAG 679-1) and *Zygnema circumcarinatum *Czurda (SAG 698-1a) were obtained by isopycnic centrifugation of total cellular DNAs in CsCl-bisbenzimide gradients [36]. A random clone library was prepared from each algal cpDNA fraction as follows. DNA was sheared by nebulization and 1,500–2,000-bp fragments were recovered by electroelution after agarose gel electrophoresis. These fragments were treated with *E. coli *Klenow fragment and T7 DNA polymerase and cloned into the SmaI site of Bluescript II KS+ or into ligation-ready pSMART-HCKan (Lucigen Corporation, Middleton). After filter hybridization of the clones with the original DNA used for cloning as a probe, DNA templates from positive clones were prepared with the QIAprep 96 Miniprep kit (Qiagen Inc., Canada).

### Sequence analyses

Nucleotide sequences were determined with the PRISM BigDye terminator cycle sequencing ready reaction kit (Applied Biosystems, Foster City, CA), the PRISM dGTP BigDye terminator ready reaction kit (Applied Biosystems), and the DYEnamic ET terminator cycle sequencing kit (Amersham Pharmacia Biotech, Canada) on ABI model 373 or 377 DNA sequencers (Applied Biosystems), using T3 and T7 primers as well as oligonucleotides complementary to internal regions of the plasmid DNA inserts. Genomic regions not represented in the clones analyzed were sequenced from PCR-amplified fragments. Sequences were assembled using SEQUENCHER 4.1.1 (Gene Codes Corporation, Ann Arbor, MI) and analyzed using the Genetics Computer Group (Madison, WI) software (version 10.3) package. Protein-coding and rRNA genes were identified by BLAST searches [37] of the nonredundant database at the National Center for Biotechnology Information, and tRNA genes were found using tRNAscan-SE [38]. Repeated sequence elements were searched using REPuter [25]. The GRIMM web server [39] was used to infer the number of gene permutations by inversions. Genes within copy A of the *Chaetosphaeridium *and *Marchantia *IRs were excluded in these gene order analyses. Pairwise comparisons of genome sequences were carried out using PipMaker [24].

## Abbreviations

cpDNA, chloroplast DNA; IR, inverted repeat; ORF, open reading frame; rRNA, ribosomal RNA.

## Authors' contributions

MT conceived and designed the study, contributed to the analysis and interpretation of the data and wrote the manuscript. CO carried out the sequencing of the *Staurastrum *and *Zygnema *chloroplast genomes. CO and CL participated in the assembly of the genome sequences. CL performed all sequence analyses and generated the figures. All authors read and approved the final manuscript.

## Figures and Tables

**Table 1:** General features of cpDNAs from *Staurastrum*, *Zygnema*, other streptophytes and *Mesostigma*

**Feature**	***Mesostigma***	***Staurastrum***	***Zygnema***	***Chaetosphaeridium***	***Marchantia***
Size^a ^(bp)					
IR	6,057	-	-	12,431	10,058
SSC	22,619	-	-	17,639	19,813
LSC	83,627	-	-	88,682	81,095
Genome	118,360	157,089	165,372	131,183	121,024
					
A+T content (%)	69.9	67.5	68.9	70.4	71.2
					
Gene content^b^	136	121	125	125	120
					
Introns					
Group I	0	1	1	1	1
Group II					
*Cis*-spliced	0	6	11	16	18
*Trans*-spliced	0	1	1	1	1

^a ^Because *Staurastrum *and *Zygnema *cpDNAs lack an IR, only the genome size is given for each of these cpDNAs. SSC, small single-copy region; LSC, large single-copy region.

^b ^Unique ORFs, intron ORFs and pseudogenes were not taken into account. Note that *Chaetosphaeridium tufA *was considered to be a functional gene.

**Table 2:** Proportion and base composition of coding sequences, intergenic spacers and introns in *Staurastrum*, *Zygnema*, *Chaetosphaeridium *and *Marchantia *cpDNAs

**Sequences**	***Staurastrum***	***Zygnema***	***Chaetosphaeridium***	***Marchantia***
Coding sequences^a^				
Fraction of genome (%)	51.4	50.8	67.5	69.9
A+T content (%)	65.1	63.0	66.1	67.7
				
Intergenic spacers				
Fraction of genome (%)	42.0	42.2	23.1	19.3
A+T content (%)	70.0	75.7	79.8	80.6
Average size (bp)	536	546	223	178
				
Introns				
Fraction of genome (%)	6.6	7.0	9.4	10.7
A+T content (%)	70.8	71.1	77.6	76.8
Average size (bp)	1,298	892	686	650

^a ^Unique ORFs and intron ORFs were not considered to be coding sequences.

**Table 3:** Differences between the gene repertoires of *Staurastrum*, *Zygnema*, *Chaetosphaeridium *and *Marchantia *cpDNAs

**Gene^a^**	***Staurastrum***	***Zygnema***	***Chaetosphaeridium***	***Marchantia***
*chlI*	+	+	+	-
*cysA*	-	+	-	+
*cysT*	-	+	-	+
*odpB*	+	+	+	-
*rpl5*	-	+	+	-
*rpl22*	+	-	+	+
*rpl32*	-	-	+	+
*rps16*	+	+	+	-
*tufA*	-	-	+^c^	-
*ycf62*	+	+	+	-
*trnP(ggg)*	-	+	+	-^b^
*trnS(cga)*	-^b^	+	-	-

^a ^Only the conserved genes that are missing in one or more chloroplast genomes are indicated. Plus and minus signs denote the presence and absence of genes, respectively.

^b ^Pseudogenes.

^c ^*Chaetosphaeridium tufA *could be a pseudogene because its sequence is highly divergent from those of other green plants.

**Table 4:** Number of inversions accounting for the gene rearrangements between *Staurastrum*, *Zygnema*, *Chaetosphaeridium *and *Marchantia *cpDNAs

**Compared cpDNA**	**Number of inversions**			
	
	*Staurastrum*	*Zygnema*	*Chaetosphaeridium*	*Marchantia*
*Staurastrum*	-	59	45	35
*Zygnema*	-	-	59	54
*Chaetosphaeridium*	-	-	-	13
*Marchantia*	-	-	-	-

**Table 5:** *Zygnema *cpDNA regions containing tandem repeats

**Repeat region^a^**	**Repeat unit**	**Number of units^b^**
3276 – 3360	CTTAA	17
11203 – 11242	GTAT	10
11535 – 11602	GTAT	17
14272 – 14319	CTTA	12
15765 – 15807	AGAAAG	7
17944 – 18047	GTAT	26
18110 – 18179	TAGAA	14
18184 – 18263	CTTTT	16
24490 – 24565	ATAC	19
30556 – 30597	AAGTAC	7
32429 – 32533	GTAAA	21
34907 – 35018	ATAC	28
49994 – 50038	TAGAA	9
51521 – 51580	GTAT	15
51618 – 51817	CAAA	50
55388 – 55442	CTTTA	11
59550 – 59613	TGTGTTTGTATATTTA	4
60129 – 60183	TTCTA	11
68724 – 68763	TTCT	10
73516 – 73571	CTTA	14
73876 – 73915	ATAC	10
73919 – 73954	GTAT	9
88870 – 88925	ATAC	13
90538 – 90581	GAAT	11
92651 – 92730	TATATTACAT	8
102484 – 102531	TTTTAAAT	6
103132 – 103183	AATT	13
103629 – 103676	ATAC	12
104932 – 105090	GTAT	33
106702 – 106737	GTAT	9
132449 – 132496	GTAT	12
134893 – 134932	CTTA	10
140237 – 140308	TTACAATAGATT	6
143451 – 143485	TAATA	7
161662 – 161696	TTCTA	7

^a ^Only the repeat regions larger than 30 bp are indicated; their coordinates refer to [GenBank:AY958086].

^b ^The number of units was estimated by allowing one substitution per repeat unit.

## References

[B1] Graham LE, Cook ME, Busse JS (2000). The origin of plants: body plan changes contributing to a major evolutionary radiation. Proc Natl Acad Sci USA.

[B2] Kenrick P, Crane PR (1997). The origin and early evolution of plants on land. Nature.

[B3] Sanderson MJ, Thorne JL, Wikstrom N, Bremer K (2004). Molecular evidence on plant divergence times. Am J Bot.

[B4] Lewis LA, McCourt RM (2004). Green algae and the origin of land plants. Am J Bot.

[B5] Bremer K, Humphries CJ, Mishler BD, Churchill SP (1987). On cladistic relationships in green plants. Taxon.

[B6] Mattox KR, Stewart KD, Irvine DEG, John DM (1984). Classification of the green algae: a concept based on comparative cytology. The Systematics of the Green Algae.

[B7] Bhattacharya D, Weber K, An SS, Berning-Koch W (1998). Actin phylogeny identifies *Mesostigma viride *as a flagellate ancestor of the land plants. J Mol Evol.

[B8] Karol KG, McCourt RM, Cimino MT, Delwiche CF (2001). The closest living relatives of land plants. Science.

[B9] Marin B, Melkonian M (1999). Mesostigmatophyceae, a new class of streptophyte green algae revealed by SSU rRNA sequence comparisons. Protist.

[B10] Martin W, Rujan T, Richly E, Hansen A, Cornelsen S, Lins T, Leister D, Stoebe B, Hasegawa M, Penny D (2002). Evolutionary analysis of *Arabidopsis*, cyanobacterial, and chloroplast genomes reveals plastid phylogeny and thousands of cyanobacterial genes in the nucleus. Proc Natl Acad Sci USA.

[B11] Lemieux C, Otis C, Turmel M (2000). Ancestral chloroplast genome in *Mesostigma viride *reveals an early branch of green plant evolution. Nature.

[B12] Turmel M, Otis C, Lemieux C (2002). The complete mitochondrial DNA sequence of *Mesostigma viride *identifies this green alga as the earliest green plant divergence and predicts a highly compact mitochondrial genome in the ancestor of all green plants. Mol Biol Evol.

[B13] Martin W, Deusch O, Stawski N, Grunheit N, Goremykin V (2005). Chloroplast genome phylogenetics: why we need independent approaches to plant molecular evolution. Trends Plant Sci.

[B14] Turmel M, Ehara M, Otis C, Lemieux C (2002). Phylogenetic relationships among streptophytes as inferred from chloroplast small and large subunit rRNA gene sequences. J Phycol.

[B15] Chapman RL, Waters DA (2002). Green algae and land plants – an answer at last?. J Phycol.

[B16] Qiu YL, Palmer JD (1999). Phylogeny of early land plants: insights from genes and genomes. Trends Plant Sci.

[B17] McCourt RM, Delwiche CF, Karol KG (2004). Charophyte algae and land plant origins. Trends Ecol Evol.

[B18] Turmel M, Otis C, Lemieux C (2002). The chloroplast and mitochondrial genome sequences of the charophyte *Chaetosphaeridium globosum *: insights into the timing of the events that restructured organelle DNAs within the green algal lineage that led to land plants. Proc Natl Acad Sci USA.

[B19] Manhart JR, Hoshaw RW, Palmer JD (1990). Unique chloroplast genome in *Spirogyra maxima *(Chlorophyta) revealed by physical and gene mapping. J Phycol.

[B20] Bold H, Wynne MJ (1985). Introduction to the Algae.

[B21] McCourt RM, Karol KG, Bell J, Helm-Bychowski KM, Grajewka A, Wojciechowski MF, Hoshaw R (2000). Phylogeny of the conjugating green algae (Zygnemophyceae) based on *rbcL *sequences. J Phycol.

[B22] Gontcharov AA, Marin B, Melkonian M (2004). Are combined analyses better than single gene phylogenies? A case study using SSU rDNA and *rbcL *sequence comparisons in the Zygnematophyceae (Streptophyta). Mol Biol Evol.

[B23] Ohyama K, Fukuzawa H, Kohchi T, Shirai H, Sano T, Sano S, Umesono K, Shiki Y, Takeuchi M, Chang Z (1986). Chloroplast gene organization deduced from complete sequence of liverwort *Marchantia polymorpha *chloroplast DNA. Nature.

[B24] Schwartz S, Zhang Z, Frazer K, Smit A, Riemer C, Bouck J, Gibbs R, Hardison R, Miller W (2000). PipMaker: a web server for aligning two genomic DNA sequences. Genome Res.

[B25] Kurtz S, Choudhuri JV, Ohlebusch E, Schleiermacher C, Stoye J, Giegerich R (2001). REPuter: the manifold applications of repeat analysis on a genomic scale. Nucleic Acids Res.

[B26] Palmer JD, Osorio B, Aldrich J, Thompson WF (1987). Chloroplast DNA evolution among legumes: Loss of a large inverted repeat occurred prior to other sequence rearrangements. Curr Genet.

[B27] Strauss SH, Palmer JD, Howe GT, Doerksen AH (1988). Chloroplast genomes of two conifers lack a large inverted repeat and are extensively rearranged. Proc Natl Acad Sci USA.

[B28] Palmer JD, Bogorad L, Vasil K (1991). Plastid chromosomes: structure and evolution. The Molecular Biology of Plastids.

[B29] Wolfe KH, Morden CW, Palmer JD (1992). Function and evolution of a minimal plastid genome from a nonphotosynthetic parasitic plant. Proc Natl Acad Sci USA.

[B30] Sears BB, Chiu W-L, Wolfson R, Tsenewaki K (1995). Replication slippage as a molecular mechanism for evolutionary variation in chloroplast DNA due to deletions and insertions. Plant genome and plastome: their structure and evolution.

[B31] Dujon B (1989). Group I introns as mobile genetic elements: Facts and mechanistic speculations – A review. Gene.

[B32] Hess WR, Hoch B, Zeltz P, Hubschmann T, Kossel H, Borner T (1994). Inefficient *rpl2 *splicing in barley mutants with ribosome-deficient plastids. Plant Cell.

[B33] Hubschmann T, Hess WR, Borner T (1996). Impaired splicing of the *rps12 *transcript in ribosome-deficient plastids. Plant Mol Biol.

[B34] Jenkins BD, Kulhanek DJ, Barkan A (1997). Nuclear mutations that block group II RNA splicing in maize chloroplasts reveal several intron classes with distinct requirements for splicing factors. Plant Cell.

[B35] Vogel J, Borner T, Hess WR (1999). Comparative analysis of splicing of the complete set of chloroplast group II introns in three higher plant mutants. Nucleic Acids Res.

[B36] Turmel M, Lemieux C, Burger G, Lang BF, Otis C, Plante I, Gray MW (1999). The complete mitochondrial DNA sequences of *Nephroselmis olivacea *and *Pedinomonas minor*: two radically different evolutionary patterns within green algae. Plant Cell.

[B37] Altschul SF, Gish W, Miller W, Myers EW, Lipman DJ (1990). Basic local alignment search tool. J Mol Biol.

[B38] Lowe TM, Eddy SR (1997). tRNAscan-SE: a program for improved detection of transfer RNA genes in genomic sequence. Nucleic Acids Res.

[B39] Tesler G (2002). GRIMM: genome rearrangements web server. Bioinformatics.

[B40] Notredame C, Higgins DG, Heringa J (2000). T-Coffee: a novel method for fast and accurate multiple sequence alignment. J Mol Biol.

[B41] Livingstone CD, Barton GJ (1993). Protein sequence alignments: a strategy for the hierarchical analysis of residue conservation. Comput Appl Biosci.

